# Thoracic Radiotherapy in Limited-Stage SCLC—a Population-Based Study of Patterns of Care in Norway From 2000 Until 2018

**DOI:** 10.1016/j.jtocrr.2021.100270

**Published:** 2021-12-18

**Authors:** Gustav Graabak, Bjørn Henning Grønberg, Marie Søfteland Sandvei, Yngvar Nilssen, Tarje Onsøien Halvorsen

**Affiliations:** a Department of Clinical and Molecular Medicine, NTNU, Norwegian University of Science and Technology, Trondheim, Norway; b Department of Oncology, St Olav’s Hospital, Trondheim University Hospital, Trondheim, Norway; c Department of Public Health and Nursing, NTNU, Norwegian University of Science and Technology, Trondheim, Norway; d Department of Registration, Cancer Registry of Norway, Oslo, Norway

**Keywords:** Survival, Radiotherapy schedule, Twice-daily, Hyperfractionated, Hypofractionated, Accelerated

## Abstract

**Introduction:**

Twice-daily (BID) thoracic radiotherapy (TRT) of 45 Gy per 30 fractions is recommended for limited-stage (LS) SCLC, but most patients are treated with once-daily (OD) schedules owing to toxicity concerns and logistic challenges. An alternative is hypofractionated OD TRT of 40 to 42 Gy per 15 fractions. A randomized trial by our group indicated that TRT of 45 Gy per 30 fractions is more effective than TRT of 42 Gy per 15 fractions, and because it was not more toxic, 45 BID replaced 42 OD as the recommended schedule in Norway. The aims of this study were to evaluate to what extent BID TRT has been implemented in Norway and whether this practice change has led to improved survival.

**Methods:**

Data on all patients diagnosed with LS SCLC from 2000 until 2018 were collected from the Cancer Registry of Norway, containing nearly complete data on cancer diagnosis, radiotherapy, and survival.

**Results:**

A total of 2222 patients were identified; median age was 69 years, 51.8% were women, and 87.1% had stage II to III disease. Overall, 64.6% received TRT. The use of BID TRT increased from 1.8% (2000–2004) to 83.2% (2015–2018). Median overall survival among patients receiving curative TRT improved significantly during the study period (2000–2004: 17.9 mo, 2015–2018: 25.0 mo, *p* = 0.0023), and patients receiving 45 BID had significantly longer median overall survival than patients receiving 42 OD (BID: 26.2 mo, OD: 19.6 mo, *p* = 0.0015).

**Conclusions:**

BID TRT has replaced hypofractionated OD TRT as the standard treatment of LS SCLC in Norway which has led to a significant (*p* = 0.0023) and clinically relevant survival improvement.

## Introduction

SCLC comprises 13% to 15% of all lung cancer cases.^1^, ^2^, ^3^ Basic treatment is platinum/etoposide chemotherapy.^4^^,^^5^ Concurrent thoracic radiotherapy (TRT) improves survival when all lesions can be included in a radiotherapy field (limited-stage [LS]).^6^^,^^7^ Responders are offered prophylactic cranial irradiation (PCI), which reduces the risk of brain metastasis and improves survival.^8^

It is well documented that TRT should be administered early and concurrently with chemotherapy,^9^ but the optimal TRT-schedule is under debate. In the Intergroup 0096 trial from 1999, accelerated hyperfractionated twice-daily (BID) TRT of 45 Gy per 30 fractions (45 BID) improved survival but caused more esophagitis compared with once-daily (OD) TRT of 45 Gy per 25 fractions.^10^ Subsequent trials and population-based studies confirm that 45 BID is more effective than OD schedules,^11^, ^12^, ^13^, ^14^, ^15^ and that toxicity is lower when modern radiotherapy techniques are used and fewer lymph node stations are included in radiotherapy fields.^11^, ^12^, ^13^^,^^16^ Thus, BID TRT is best documented and most recommended for LS SCLC.^17^, ^18^, ^19^, ^20^, ^21^, ^22^ However, it is poorly implemented owing to concerns about toxicity, logistical challenges, and inconvenience for patients.^14^^,^^15^^,^^23^, ^24^, ^25^, ^26^, ^27^, ^28^, ^29^, ^30^, ^31^, ^32^ For example, only 11.3% of patients with LS SCLC in the US received BID TRT between 1999 and 2012,^14^ and a recent European survey reveal that most radiation oncologists prefer OD TRT even if the CONVERT trial failed to reveal that high-dose OD TRT is superior to 45 BID.^12^^,^^25^

Alternative schedules include hypofractionated OD TRT of 40 to 42 Gy per 15 fractions,^5^^,^^33^ and 42 Gy per 15 fractions (42 OD) used to be standard in Norway.^5^ It was considered less toxic, equally effective, and more convenient. Our group was the first to conduct a randomized trial (“HAST” trial) comparing 45 BID and 42 OD.^11^ Patients in the BID arm achieved a longer median overall survival (OS) (25.1 mo versus 18.8 mo), though the difference was not statistically significant (*p* = 0.61). Notably, the BID schedule did not cause more toxicity, and considering all other evidence, 45 BID replaced 42 OD as recommended schedule in Norwegian guidelines after the publication of our study in 2016.^11^^,^^34^

Study populations are in general healthier than many patients seen in the clinic.^35^, ^36^, ^37^ There seems to be concerns about administering BID TRT to older patients and patients with stage III disease.^14^ In a study from the Netherlands, there was no survival improvement after a change in guidelines recommending 45 BID instead of OD TRT.^23^ Thus, we performed this population-based study to evaluate to what extent BID TRT has been implemented in Norway and whether it has led to improved survival.

## Material and Methods

### Cancer Registry of Norway

The Cancer Registry of Norway (CRN) has been collecting data on all patients with cancer in Norway since the 1950s. All Norwegian health care services are obliged by law to report data to the CRN. Patient consent is not required. For lung cancer, the CRN is estimated to have a completeness of 96.9%.^38^ Main data sources are clinical notifications, pathology reports, and death certificates. The CRN has received radiotherapy data directly from all radiotherapy departments since 1997. A unique identification number of each Norwegian citizen enables data cross-linking with other national registries such as the Norwegian Patient Registry and the Cause of Death Registry. The cross-linking generates a regular update of patient’s vital status (dead, emigrated, or alive) and ensures that unreported cases are identified and registered in the CRN. The CRN did not contain chemotherapy data for the study period and does not collect treatment toxicity data.

### Patient Selection

**Figure 1:**
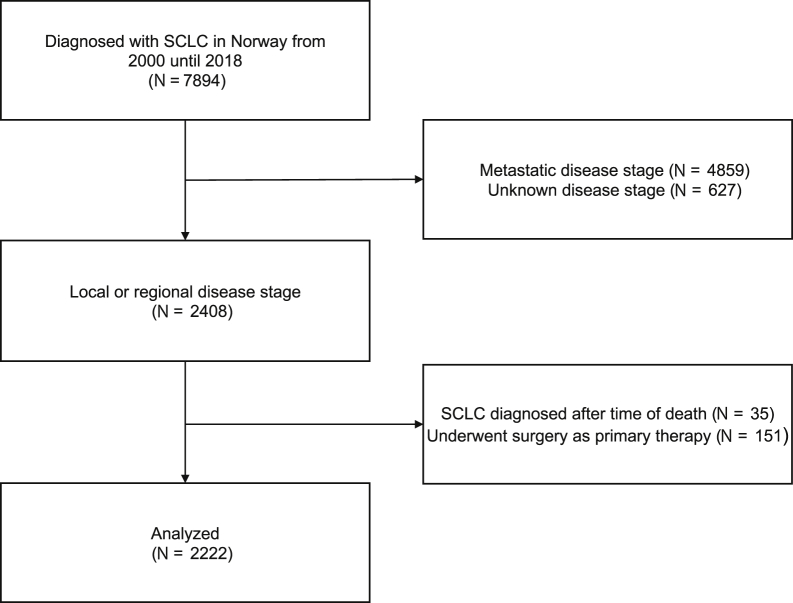
Patient selection.

Eligible patients had (1) confirmed SCLC, (2) were diagnosed from January 1, 2000 until December 31, 2018, and (3) had LS, defined as local (TNM stage I) or regional disease (TNM stage II–III). Patients who underwent surgery as primary treatment or were diagnosed postmortem were excluded (Fig. 1).

### Patient and Treatment Characteristics

Age, sex, diagnosis (SCLC or combined SCLC and NSCLC), disease stage (local, regional, metastatic, or unknown), year of diagnosis, surgery, radiotherapy (body region, fraction dose, number of fractions, total dose, and days from diagnosis until start of radiotherapy), and survival status (dead, alive, or emigrated) were extracted from the CRN. Cutoff date for survival status was June 30, 2019.

### Thoracic Radiotherapy

The first TRT after diagnosis of SCLC was the main variable. Schedules with a total dose of greater than or equal to 42 Gy were classified as curative, whereas schedules with a total dose of less than 42 Gy were classified as palliative (hypofractionated TRT of 40 Gy per 15 fractions has not been used in Norway).

The schedule of TRT of 45 Gy per 30 fractions, two fractions per day, was classified as 45 BID and TRT of 42 Gy per 15 fractions, one fraction per day, as 42 OD. High-dose BID TRT of 60 Gy per 40 fractions, used in a randomized trial, was classified as 60 BID.^22^ Schedules of 45 to 66 Gy per three fractions, 55 Gy per five fractions, and 56 Gy per eight fractions were classified as stereotactic body radiation therapy (SBRT). Other schedules with curative doses were pooled as one group in the analyses.

The CRN contains data on delivered and not planned radiotherapy doses. Therefore, we classified all patients receiving less than or equal to 30 fractions of 1.5 Gy as 45 BID, and patients receiving 31 to 40 fractions of 1.5 Gy as 60 BID because we assumed these to be the planned schedules. Patients receiving 2.8 Gy fractions were classified as 42 OD because this was the only schedule with this fraction size used for LS SCLC in Norway during the study period. The schedules were classified as not completed if more than two treatments days were omitted.

Allowing one week from diagnosis until start of chemotherapy, it takes a minimum of 70 days to administer four platinum/etoposide-courses.^34^ Consequently, TRT starting within 70 days after diagnosis was considered concurrent chemoradiotherapy and TRT starting later was considered sequential therapy.

### Prophylactic Cranial Irradiation

Whole brain irradiation of 25 Gy per 10 fractions (current recommendation) or 30 Gy per 15 fractions (previous recommendation) were classified as PCI.^34^ In Norway, schedules of 30 Gy per 10 fractions and 20 Gy per five fractions are used for whole brain irradiation of brain metastases.

### Statistical Considerations

The study period was divided into four time periods: 2000 to 2004 (before HAST trial), 2005 to 2010 (HAST enrolment period), 2011 to 2014 (HAST results presented at ASCO 2012), and 2015 to 2018 (HAST results published in 2016).

Implementation of BID TRT was reported as the proportion receiving BID TRT of patients receiving curative TRT. OS between time periods was compared among patients receiving curative TRT and between patients receiving 45 BID and 42 OD. Sensitivity analyses included only the patients who completed 45 BID and 42 OD.

OS was defined as time from diagnosis until time of death from any cause and estimated using the Kaplan-Meier method. OS was compared using the log-rank test for univariable analyses and the Cox proportional hazard method for multivariable analyses. Cox models were adjusted for baseline characteristics (age [continuous variable], sex, disease stage), and TRT (all patients; curative/palliative/no TRT, patients receiving curative TRT; TRT-schedule, time-to-start of TRT). We expected the use of BID TRT to change significantly over time and did not adjust for time, but rather performed sensitivity analyses comparing OS in different periods for patients receiving 45 BID and 42 OD. Patients lost to follow-up were excluded from survival analyses. The independent samples *t*-test and the chi-square test were used for group comparisons. A *p* value of less than or equal to 0.05 was considered statistically significant. Statistical analyses were performed using IBM SPSS Statistics version 26.

## Results

### Patient Characteristics

From 2000 until 2018, a total of 2408 patients were diagnosed with local or regional stage SCLC in Norway. We excluded patients who underwent surgery (n = 151) or were diagnosed postmortem (n = 35) and analyzed the remaining 2222 patients (Fig. 1). Median age at diagnosis was 69 years (interquartile range [IQR]: 61–75). There were 51.8% women, 0.9% had combined SCLC and NSCLC, and 87.1% had regional stage disease. The proportion of women increased (2000–2004: 46.2%, 2015–2018: 58.4%), whereas the proportion of patients with local stage disease decreased (2000–2004: 18.7%, 2015–2018: 4.9%) (Table 1).

**Table 1:** Baseline and Treatment Characteristics for Patients Diagnosed With Limited-Stage SCLC in Norway From 2000 Until 2018

Baseline and Treatment Characteristics		Y of Diagnosis				
		2000–2004 (n = 513)	2005–2010 (n = 651)	2011–2014 (n = 543)	2015–2018 (n = 515)	All Patients (N = 2222)
Age	Median number of y (IQR)	69 (60–75)	68 (61–75)	68 (61–75)	70 (63–75)	69 (61–75)
Sex	Women, n (%)	237 (46.2)	340 (52.2)	272 (50.1)	301 (58.4)	1150 (51.8)
	Men, n (%)	276 (53.8)	311 (47.8)	271 (49.9)	214 (41.6)	1072 (48.2)
Diagnosis	SCLC, n (%)	512 (99.8)	645 (99.1)	538 (99.1)	507 (98.4)	2202 (99.1)
	Combined SCLC and NSCLC, n (%)	1 (0.2)	6 (0.9)	5 (0.9)	8 (1.6)	20 (0.9)
Stage	Local, n (%)	96 (18.7)	94 (14.4)	72 (13.3)	25 (4.9)	287 (12.9)
	Regional, n (%)	417 (81.3)	557 (85.6)	471 (86.7)	490 (95.1)	1935 (87.1)
TRT	Curative; ≥42 Gy, n (%)	284 (55.4)	357 (54.8)	319 (58.7)	251 (48.7)	1211 (54.5)
	Palliative; <42 Gy, n (%)	31 (6.0)	58 (8.9)	63 (11.6)	73 (14.2)	225 (10.1)
	No TRT, n (%)	198 (38.6)	236 (36.3)	161 (29.7)	191 (37.1)	786 (35.4)
PCI	Yes, n (%)	157 (30.6)	256 (39.3)	258 (47.5)	233 (45.2)	904 (40.7)
	No, n (%)	356 (69.4)	395 (60.7)	285 (52.5)	282 (54.8)	1318 (59.3)
	Median number of d from diagnosis of SCLC until start (IQR)	188 (163–216)	146 (127–174)	132 (118–148)	127 (116–140)	139 (123–169)

IQR, interquartile range; PCI, prophylactic cranial irradiation; TRT, thoracic radiotherapy.

Among patients receiving 45 BID or 42 OD, there were more women (BID: 56.9%, OD: 48.0%, *p* = 0.0077) and more patients with regional stage disease (BID: 90.1%, OD: 83.2%, *p* = 0.0037) in the 45 BID group. There was no difference in age (BID: median 66 y [IQR: 60–71], OD: median 65 y [IQR: 59–72], *p* = 0.95) (Table 2).

**Table 2:** Baseline and Treatment Characteristics for Patients Receiving TRT of 45 Gy Per 30 Fractions and 42 Gy Per 15 Fractions

Baseline and Treatment Characteristics		45 Gy per 30 Fractions (n = 313)	42 Gy per 15 Fractions (n = 792)	*p*
Age	Median number of y (IQR)	66 (60–71)	65 (59–72)	0.95
Sex	Women, n (%)	178 (56.9)	380 (48.0)	
	Men, n (%)	135 (43.1)	412 (52.0)	0.0077
Stage	Local, n (%)	31 (9.9)	133 (16.8)	
	Regional, n (%)	282 (90.1)	659 (83.2)	0.0037
Time form diagnosis of SCLC until start of TRT	≤70 d, n (%)	295 (94.2)	507 (64.0)	
	>70 d, n (%)	18 (5.8)	285 (36.0)	<0.001
	Median number of d (IQR)	42 (35–52)	61 (44–83)	<0.001
TRT	Completed as planned, n (%)	307 (98.1)	771 (97.3)	0.48
PCI	Yes, n (%)	232 (74.1)	508 (64.1)	
	No, n (%)	81 (25.9)	284 (35.9)	0.015
Time from diagnosis of SCLC until start of PCI	Median number of d (IQR)	130 (118–144)	151 (130–182)	<0.001
Treatment related deaths	Death ≤30 d after start of TRT, n (%)	4 (1.3)	7 (0.9)	0.55
	Death ≤90 d after start of TRT, n (%)	11 (3.5)	40 (5.1)	0.27

IQR, interquartile range; PCI, prophylactic cranial irradiation; TRT, thoracic radiotherapy.

### Thoracic Radiotherapy

Overall, 1436 patients (64.6%) received TRT and the proportion varied between 61.4% and 70.3% in time periods (Table 1). Of these, 84.3% received curative TRT, and this proportion decreased during the study period (2000–2004: 90.2%, 2015–2018: 77.5%). Median time from diagnosis until first fraction of curative TRT decreased over time (2000–2004: 71 d [IQR: 57–97], 2015–2018: 41 d [IQR: 34–51]) (Table 3).

**Table 3:** Schedules for Those Receiving Potentially Curative TRT

Thoracic Radiotherapy	Y of Diagnosis				
	2000–2004 (n = 284)	2005–2010 (n = 357)	2011–2014 (n = 319)	2015–2018 (n = 251)	All Patients (N = 1211)
% of all patients who received TRT	90.2	86.0	83.5	77.5	84.3
42 Gy/15 fractions, n (%)	269 (94.7)	291 (81.5)	197 (61.8)	35 (13.9)	792 (65.4)
45 Gy/30 fractions, n (%)	5 (1.8)	53 (14.8)	95 (29.8)	160 (63.7)	313 (25.8)
60 Gy/40 fractions, n (%)	0 (0.0)	0 (0.0)	7 (2.2)	49 (19.5)	56 (4.6)
Other schedules of ≥42 Gy^a^, n (%)	10 (3.5)	10 (2.8)	13 (4.1)	2 (0.8)	35 (2.9)
Stereotactic body radiotherapy^b^, n (%)	0 (0.0)	3 (0.8)	7 (2.2)	5 (2.0)	15 (1.2)
TRT initiated ≤70 d after diagnosis of SCLC, n (%)	139 (48.9)	254 (71.1)	258 (80.9)	226 (90.0)	877 (72.4)
TRT initiated >70 d after diagnosis of SCLC, n (%)	145 (51.1)	103 (28.9)	61 (19.1)	25 (10.0)	334 (27.6)
Median number of d from diagnosis of SCLC until start of TRT (IQR)	71 (57–97)	53 (41–76)	48 (39–63)	41 (34–51)	52 (41–74)

IQR, interquartile range; TRT, thoracic radiotherapy.

a A total of 1.7 to 3.4 Gy fractions to doses of 42 to 70 Gy (excluding 42 Gy per 15 fractions).

b A total of 45 Gy per three fractions, 54 Gy per three fractions, 55 Gy per five fractions, 56 Gy per eight fractions, or 66 Gy per three fractions.

**Figure 2:**
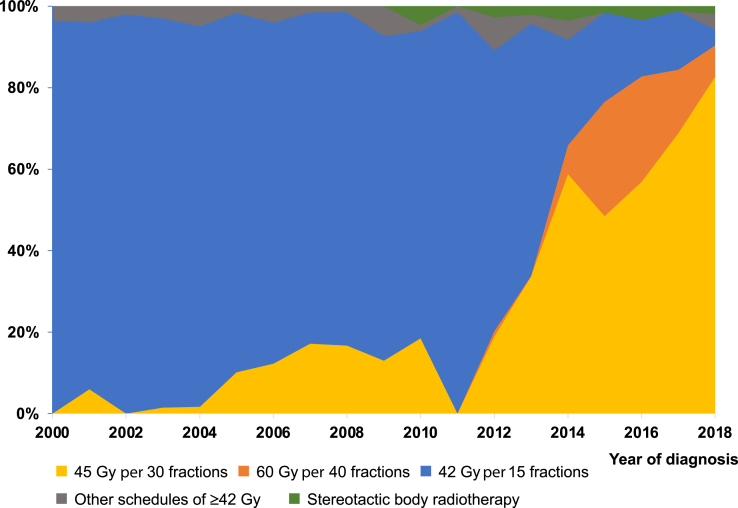
Thoracic radiotherapy schedules of greater than or equal to 42 Gy administered to patients diagnosed with limited-stage SCLC in Norway from 2000 until 2018.

The most common schedules of curative TRT were 42 OD (65.4%) and 45 BID (25.8%). The use of 45 BID increased rapidly between 2011 and 2014 (2011: 0.0%, 2012: 18.9%, 2013: 33.7%, 2014: 58.8%) and considerably from the beginning until the end of the study period (2000–2004: 1.8%, 2015–2018: 63.7%) (Fig. 2). The 60 BID schedule was mainly used in the last period as part of a randomized trial (2015–2018: 19.5%, other periods: ≤2.2%).^22^ In total, BID TRT comprised 83.2% of all curative TRT in the last period (2015–2018) and 90.4% in 2018. Correspondingly, the use of 42 OD decreased (2000–2004: 94.7%, 2015–2018: 13.9%) (Table 3).

A larger proportion receiving 45 BID started TRT less than or equal to 70 days after diagnosis than those receiving 42 OD (BID: 94.2%, OD: 64.0%, *p* < 0.001), and median time from diagnosis until start of TRT was shorter for 45 BID (BID: 42 d [IQR: 35–52], OD: 61 d [44–83], *p* < 0.001). The proportions completing TRT as planned were similar (BID: 98.1%, OD: 97.3%, *p* = 0.48) (Table 2).

A few patients received SBRT (1.2%) and other schedules of greater than or equal to 42 Gy (2.9%). Among patients receiving the latter, mean total dose was 48.5 Gy, nine patients (25.7%) received 2 Gy fractions to doses of 42 to 70 Gy, whereas 26 patients (74.3%) received 1.7 to 3.4 Gy fractions to doses of 42 to 56 Gy (excluding 42 Gy per 15 fractions and schedules of 2 Gy fractions).

### Prophylactic Cranial Irradiation

Overall, 40.7% received PCI, and the proportion increased from 30.6% between 2000 and 2004 to 45.2% between 2015 and 2018 (Table 1). PCI was given to a larger proportion of patients receiving 45 BID than 42 OD (BID: 74.1%, OD: 64.1%, *p* = 0.015) (Table 2).

### Survival

#### All Patients

Seven patients were lost to follow-up and excluded from survival analyses. Overall, median OS was 13.0 months (95% confidence interval [CI]: 12.3–13.6) and increased significantly during the study period (median OS 2000–2004: 10.8 mo [95% CI: 9.8–11.9], 2005–2010: 12.3 mo [95% CI: 11.3–13.4], 2011–2014: 14.6 mo [95% CI: 12.9–16.2], 2015–2018: 14.4 mo [95% CI: 12.7–16.0], *p* < 0.001).

#### Thoracic Radiotherapy

Median OS among patients receiving TRT was 19.1 months (95% CI: 18.1–20.1). Patients receiving curative TRT had significantly longer OS than patients receiving palliative TRT or no TRT (median OS ≥42 Gy: 21.3 mo [95% CI: 20.0–22.5], <42 Gy: 11.1 mo [95% CI: 9.7–12.4], no TRT: 4.5 mo [95% CI: 3.8–5.3], *p* < 0.001). Patients receiving curative TRT initiated less than or equal to 70 days after diagnosis lived significantly longer than patients who started later (median OS ≤70 d: 22.3 mo [95% CI: 20.5–24.2], >70 d: 19.6 mo [95% CI: 17.6–21.5], *p* = 0.0031). Patients receiving 60 BID had significantly longer OS than patients treated with other curative schedules (median OS 60 BID: 34.4 mo [95% CI: 17.9–50.9], 45 BID: 26.2 mo [95% CI: 21.6–30.8], 42 OD: 19.6 mo [95% CI: 18.3–20.9], SBRT: 29.0 mo [95% CI: 15.8–42.2], other schedules of ≥42 Gy: 21.0 mo [95% CI: 12.6–29.4], *p* < 0.001). Patients receiving 45 or 60 BID had a longer survival compared with patients receiving any OD TRT (median OS BID TRT: 27.2 mo [95% CI: 23.2–31.3], OD TRT: 19.9 mo [95% CI: 18.6–21.2], *p* < 0.001)

**Figure 3:**
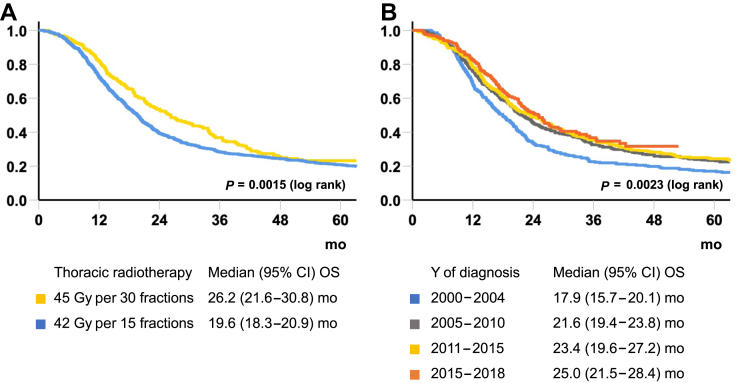
Overall survival according to (*A*) TRT of 45 Gy per 30 fractions and 42 Gy per 15 fractions, and (*B*) time period for patients receiving TRT of greater than or equal to 42 Gy. CI, confidence interval; OS, overall survival; TRT, thoracic radiotherapy.

Patients receiving 45 BID had significantly longer survival than patients receiving 42 OD (median OS 45 BID: 26.2 mo [95% CI: 21.6–30.8], 42 OD 19.6 mo [95% CI: 18.3–20.9], *p* = 0.0015) (Fig. 3*A*), also among those who started TRT less than or equal to 70 days after diagnosis (median OS 45 BID: 26.3 mo [95% CI: 21.6–30.9], 42 OD: 19.8 mo [95% CI: 18.0–21.5], *p* = 0.012), and in sensitivity analysis excluding those who did not complete TRT as planned (median OS 45 BID: 26.3 mo [95% CI: 21.9–30.7], 42 OD: 19.9 mo [95% CI: 18.6–21.2], *p* = 0.020).

There was no significant difference in OS among those receiving 45 BID in different time periods (2000–2004 excluded owing to few patients, median OS 2005–2010: 33.7 mo [95% CI: 26.0–41.4], 2011–2014: 26.3 mo [95% CI: 16.9–35.6], 2015–2018: 22.8 mo [95% CI: 18.9–26.7], *p* = 0.52). OS from 42 OD increased during the first periods but was shortest in the last period (median OS 2000–2004: 17.9 mo [95% CI: 15.7–20.1], 2005–2010: 20.2 mo [95% CI: 18.0–22.4], 2011–2014: 22.3 mo [95% CI: 18.2–26.4], 2015–2018: 16.6 mo [95% CI: 11.6–21.6], *p* = 0.036). In sensitivity analyses, KM curves indicated that OS from 45 BID was superior or similar to 42 OD in all time periods, but numbers were low in some time periods, precluding firm conclusions (data not shown).

Overall, median OS improved significantly during the study period for patients receiving curative TRT (median OS 2000–2004: 17.9 mo [95% CI: 15.7–20.1], 2015–2018: 25.0 mo [95% CI: 21.5–28.4], *p* = 0.0023) (Fig. 3*B*).

#### Prophylactic Cranial Irradiation

Among patients receiving curative TRT, those who received PCI lived longer than those who did not (median OS PCI: 26.2 mo [95% CI: 24.1–28.4], no PCI: 12.6 mo [95% CI: 11.5–13.7], *p* < 0.001).

#### Multivariable Analyses of Overall Survival

In multivariable survival analysis of all patients, palliative TRT (hazard ratio [HR]: 2.00 [95% CI: 1.71–2.33], *p* < 0.001) and no TRT (HR: 3.96 [95% CI: 3.56–4.40], *p* < 0.001) were associated with shorter OS than curative TRT. Age (HR: 1.03 [95% CI: 1.02–1.03], *p* < 0.001), sex (men versus women; HR: 1.17 [95% CI: 1.07–1.29], *p* < 0.001), and disease stage (regional stage versus local stage; HR: 1.43 [95% CI: 1.25–1.64], *p* < 0.001) were independent negative prognostic factors.

In the multivariable survival analysis of patients receiving curative TRT, 60 BID was associated with longer OS than 45 BID (HR: 0.62 [95% CI: 0.41–0.94], *p* = 0.024), whereas 42 OD resulted in shorter OS than 45 BID (HR: 1.23 [95% CI: 1.04–1.45], *p* = 0.015). Age (HR: 1.03 [95% CI: 1.02–1.04], *p* < 0.001) and disease stage (regional stage versus local stage; HR: 1.33 [95% CI: 1.11–1.59], *p* = 0.0019) were independent negative prognostic factors. Sex and time-to-TRT (≤70 d or >70 d) were not significantly associated with OS, but there was a trend toward shorter OS among men (HR: 1.12 [95% CI: 0.99–1.27], *p* = 0.083) and those starting TRT more than 70 days after diagnosis (HR: 1.14 [95% CI: 0.98–1.31], *p* = 0.083) (Table 4). When entering time-to-TRT as a continuous variable (d) in the model, 42 OD still resulted in shorter OS than 45 BID (HR: 1.24 [95% CI: 1.05–1.46], *p* = 0.010).

**Table 4:** Multivariable Analyses of Overall Survival for Patients Receiving TRT Schedules of Greater Than or Equal to 42 Gy

Baseline and Treatment Characteristics		No. of Cases	Hazard Ratio	95% CI	*p*
Age	Per y	1206	1.03	1.02–1.04	<0.001
Sex	Women	612	1	—	—
	Men	594	1.12	0.99–1.27	0.083
Stage	Local	182	1	—	—
	Regional	1024	1.33	1.11–1.59	0.0019
TRT	45 Gy per 30 fractions	311	1	—	—
	60 Gy per 30 fractions	56	0.62	0.41–0.94	0.024
	42 Gy per 15 fractions	789	1.23	1.04–1.45	0.015
	Other schedules of ≥42 Gy	35	0.95	0.63–1.44	0.82
	Stereotactic body radiotherapy	15	1.05	0.59–1.86	0.88
Time from diagnosis of SCLC until start of TRT	≤70 d	874	1	—	—
	>70 d	332	1.14	0.98–1.31	0.083

CI, confidence interval; TRT, thoracic radiotherapy.

## Discussion

In this population-based study, we found that use of 45 BID TRT increased from 1.8% between 2000 and 2004 to 63.7% between 2015 and 2018, and 45 BID has replaced 42 OD as the standard TRT-schedule in treatment of LS SCLC in Norway. When including the 60 BID schedule administered through a trial, 83.2% received BID TRT in the last study period (2015–2018), and 90.4% in 2018. Patients receiving 45 BID had significantly longer median OS than patients receiving 42 OD, both in univariable (26.2 mo versus 19.6 mo, *p* = 0.0015) and multivariable analyses (OD versus BID; HR: 1.23 [95% CI: 1.04–1.45]). Interestingly, the difference in median OS is almost identical to the difference observed in our randomized trial comparing these TRT-schedules (25.1 mo versus 18.8 mo).^11^ Median OS improved significantly for patients receiving curative TRT during the study period (25.0 mo versus 17.9 mo, *p* = 0.0023).

To our knowledge, the observed implementation of BID TRT in Norway is the highest reported in population-based studies of LS SCLC. In other studies, implementation rates have been between 2% and 49%,^14^^,^^15^^,^^23^, ^24^, ^25^, ^26^, ^27^, ^28^, ^29^, ^30^, ^31^, ^32^ though only two studies are based on data from registries comparable with the CRN,^14^^,^^23^ and few have investigated patterns of practice over several years.^14^^,^^15^^,^^23^^,^^24^ A study extracting data from the U.S. National Cancer Data Base reported that 11.3% of patients receiving TRT-doses of greater than or equal to 45 Gy received 45 BID in the United States between 1999 and 2012.^14^ Another study reporting data from the Netherlands Cancer Registry found an increased use of BID TRT after the schedule was recommended in national guidelines (2010–2012: 13%, 2013–2014: 36%).^23^ Furthermore, a European survey among 198 radiation oncologists revealed an increased use of BID TRT after the results of the CONVERT trial were published (32% before and 42% after publication), but most still preferred OD TRT, even though 66 Gy per 33 fractions OD failed to reveal superiority over 45 BID in the CONVERT trial.^12^^,^^25^ A survey among Canadian radiation oncologists revealed that hypofractionated OD TRT of 40 to 45 Gy per 15 fractions was the most preferred TRT-schedule in 2015 (40%).^27^

The observed 26.2 months median OS from 45 BID is comparable with previous trials (22.6–33.6 mo) and population-based series (21.5–27 mo).^10^, ^11^, ^12^^,^^14^^,^^22^^,^^23^^,^^33^^,^^39^, ^40^, ^41^ The observed 19.6 months median OS among patients receiving 42 OD is also similar to previous reports (clinical trials: 18.8–21.2 mo, population-based series: 14.7–28.1 mo).^11^^,^^39^^,^^42^, ^43^, ^44^, ^45^ The 6.6 months benefit in median OS favoring 45 BID is comparable with the 6.3 months difference in our previous trial,^11^ and results from a retrospective single institution study (5 mo benefit in median OS from 45 BID versus 40 OD).^39^ Thus, our study adds further evidence showing that 45 BID is superior to hypofractionated 40 to 42 OD. However, a recent Chinese randomized phase 2 trial (n = 182) suggests that high-dose hypofractionated OD TRT of 65 Gy per 26 fractions improve progression-free survival compared with 45 BID.^40^

OS among the few patients receiving SBRT and other schedules of greater than or equal to 42 Gy were comparable with OS from the more common TRT-schedules, but the numbers of these patients were low. The longest median OS was observed in patients receiving 60 BID (34.4 mo), but these patients were enrolled in a trial,^22^ and might have been more selected than other patients in our cohort. Whether the 60 BID schedule improves survival on a population level remains to be seen.

In the study using data from the U.S. National Cancer Data Base, survival was significantly longer among patients receiving 45 BID than those receiving OD TRT-schedules (median OS 22.1 mo versus 17.2–19.5 mo),^14^ whereas the increasing use of BID TRT in The Netherlands did not lead to improved survival (median OS 2010: 22 mo, 2014: 21 mo).^23^ However, there have been toxicity concerns, and patients older than 70 years and those with higher disease stage were less likely to receive BID TRT in the US and less likely to receive concurrent chemoradiotherapy in The Netherlands.^14^^,^^23^ In contrast, the implementation rate is much higher in our study, and one might speculate that the improved survival is owing to the fact that most elderly and most stage III patients also received BID TRT in our last study period, possibly suggesting that these patients benefit the most from BID TRT. However, these studies are not necessarily comparable with ours. Other OD TRT-schedules were used in the Netherlands, and hypofractionated TRT of 40 to 42 Gy per 15 fractions was not used.^23^

We used a condensed staging system (local, regional, metastatic, and unknown) to identify patients with LS SCLC. This might not completely correlate with definition of LS in other studies which has changed over time and varies between studies. However, patients with extensive-stage SCLC do not receive TRT of greater than or equal to 42 Gy in Norway. The CRN does not contain chemotherapy data, but four to five courses of platinum/etoposide have been standard regimen for LS SCLC in Norway since 2002.^5^ After 45 BID was recommended, 42 OD might have been offered to marginally fit patients, but information about performance status and co-morbidity are not included in the CRN. Indeed, OS from 42 OD was lower in the last time period (2015–2018: 16.6 mo, 2011–2014: 22.3 mo), but as these patients comprise a negligible proportion of patients receiving 42 OD (2015–2018: 4.4%), we believe that the impact on the overall comparison with 45 BID patients is minimal. Accurate comparison of 5-year survival was not possible as these data are immature for patients receiving 45 BID in the last period. We did not adjust for use of PCI because this is closely correlated to response of TRT, and thus, possibly TRT-schedule. The multivariable model was not adjusted for time period, but in sensitivity analyses, we found that OS from 45 BID was superior or similar to 42 OD in all periods, indicating that TRT-schedule, and not the effect of time or time-to-treatment start, is the reason for the survival improvement.

Positron emission tomography-computed tomography (PET-CT) for staging of LS SCLC was implemented in 2014 through our recent trial.^22^ PET-CT is superior to conventional CT for disease staging and improves ability to detect metastases to normal sized lymph,^46^ which might explain why the proportions with local disease and those receiving curative TRT decreased in the last time period. Defining radiotherapy fields according to PET-CT images reduces radiotherapy target volumes and normal tissue irradiation in many cases and sometimes ensures inclusion of lymph node metastases not included when applying elective nodal irradiation.^47^ Thus, it is possible that implementing PET-CT for staging has contributed to the improved survival over time.

The major strength of our study is the wide implementation of BID TRT which enabled evaluation of BID TRT in a population-based setting, and comparison of survival from 45 BID and 42 OD. We used data from a nationwide registry with high level of completeness of lung cancer cases and radiotherapy data covering a broader time period than previous studies of TRT in LS SCLC, leading to a large number of patients with minimal loss to follow-up. Furthermore, we were able to control for important treatment characteristics affecting survival, including fractionation and timing of TRT. Finally, improved survival from 45 BID was consistently shown in both the main analysis and sensitivity analyses.

Several factors may have contributed to the large adoption of BID TRT in Norway during the last decade. First, there were no longer toxicity concerns because our trial revealed that 45 BID was equally well tolerated as the previous standard schedule, 42 OD.^11^ Although the CRN does not contain toxicity data, there was at least no difference in mortality 30 and 90 days after TRT in this study (Table 2). Second, all patients with lung cancer in Norway are treated at public hospitals, whereas BID TRT seems to be more often preferred in public/academic institutions than in private practices in the United States.^14^^,^^26^ In Norway, hospital treatment is almost free of charge (patients pay a maximum of €240 per y) and opening hours does not restrict implementation of BID TRT. Many patients need to travel a long distance and stay away from home during TRT, and BID TRT lasting three to four weeks consumes less time for patients than OD TRT lasting six to seven weeks. We found that equal proportions of patients completed 45 BID and 42 OD (45 BID: 98.1%, 42 OD: 97.3%, *p* = 0.48), indicating that 45 BID is feasible and not as inconvenient for patients as previously suggested.^25^^,^^26^. Third, a substantial proportion of the 45 BID patients in this study participated in clinical trials,^11^^,^^22^ and conduction of these trials facilitated implementation. Finally, the community of physicians treating patients with SCLC in Norway is small, and most are aware of and adhere to national guidelines. In addition to the implementation of BID TRT, this is illustrated by the recommended decrease in time until start of TRT and the increased use of PCI, both contributing to improved survival in the study period. Our study demonstrates the importance of population-based studies in evaluating implementation of a previously controversial, but well documented treatment through changes in national guidelines.

In conclusion, we found that hyperfractionated BID TRT has replaced hypofractionated OD TRT as the standard treatment of LS SCLC in Norway. The implementation rate is more than 90% and much higher than in previous reports, showing that implementing BID TRT is feasible on a population level. This change in treatment policy has led to a significant survival improvement (*p* = 0.0023), supporting the conclusion from our previous trial that BID TRT is superior to OD TRT.

## CRediT Authorship Contribution Statement

**Gustav Graabak:** Formal analysis, Writing – original draft, Writing – review & editing, Visualization.

**Bjørn H. Grønberg:** Conceptualization, Methodology, Supervision, Writing – review & editing.

**Marie S. Sandvei:** Methodology, Writing – review & editing.

**Yngvar Nilssen:** Data curation, Writing – review & editing.

**Tarje O. Halvorsen:** Conceptualization, Methodology, Supervision, Writing - original draft, Writing - review & editing.

## Approval Statement

According to Norwegian laws and regulations, this study did not require ethics committee approval, and this was confirmed by the Regional Committee for Medical Research Ethics (Central Norway, Norway).
